# A Case of Upper Gastrointestinal Bleeding Due to Metastatic High-Grade B-Cell Lymphoma Successfully Treated With Chemotherapy

**DOI:** 10.7759/cureus.24738

**Published:** 2022-05-04

**Authors:** Samuel Tanner, Elie Al Kazzi, Rabail Aslam, Gerard Isenberg, Gregory Cooper

**Affiliations:** 1 Department of Medicine, University Hospitals Cleveland Medical Center, Cleveland, USA; 2 Division of Gastroenterology and Liver Disease, Department of Medicine, University Hospitals Cleveland Medical Center, Cleveland, USA; 3 Department of Pathology, University Hospitals Cleveland Medical Center, Cleveland, USA

**Keywords:** lymphoma, large b-cell lymphoma, gastrointestinal hemorrhage, endoscopy, gastric cancer

## Abstract

Upper gastrointestinal bleeding (UGIB) is a common and potentially life-threatening condition. Metastatic disease is an exceedingly rare cause of UGIB. We report the case of a 73-year-old man with high-grade B-cell lymphoma (HGBL) who presented for the initiation of chemotherapy and was found to be acutely anemic due to UGIB. An esophagogastroduodenoscopy (EGD) revealed multiple large, discrete, ulcerated, non-circumferential, and friable masses in the stomach. Biopsies were consistent with HGBL. The patient was urgently initiated on chemotherapy with the resolution of lesions on subsequent EGD. The rate of prevalence of gastric metastases is unknown, but it should be considered in patients with active malignancy who present with signs of UGIB.

## Introduction

Upper gastrointestinal bleeding (UGIB) is a potentially life-threatening condition and peptic ulcer disease (PUD) represents its most common etiology [1]. Metastatic disease is an exceedingly rare cause of UGIB [2-4]. In this report, we present the case of a patient with recently diagnosed high-grade B-cell lymphoma (HGBL) who presented with acute upper GI bleed due to metastatic HGBL in the stomach. Treatment with chemotherapy resulted in the cessation of bleeding and resolution of metastatic gastric lesions.

This article was previously presented as a meeting abstract at the 2021 ACG Annual Scientific Meeting on October 26, 2021.

## Case presentation

**Figure 1:**
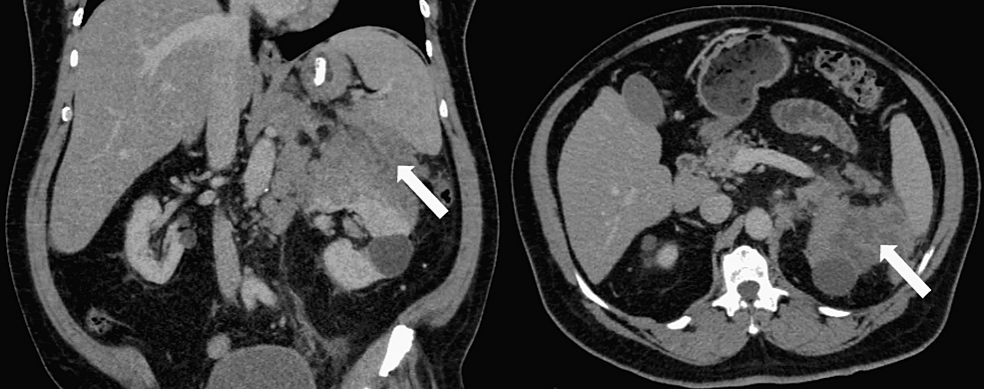
CT scan of abdomen and pelvis The images reveal a 10.8 x 8.9 x 8.2 cm irregular heterogeneous mass arising out of the superior pole of the left kidney with extension superiorly and invasion of the undersurface of the spleen. There is celiac axis adenopathy as well as retroperitoneal adenopathy and splenic hilum adenopathy CT: computed tomography

**Figure 2:**
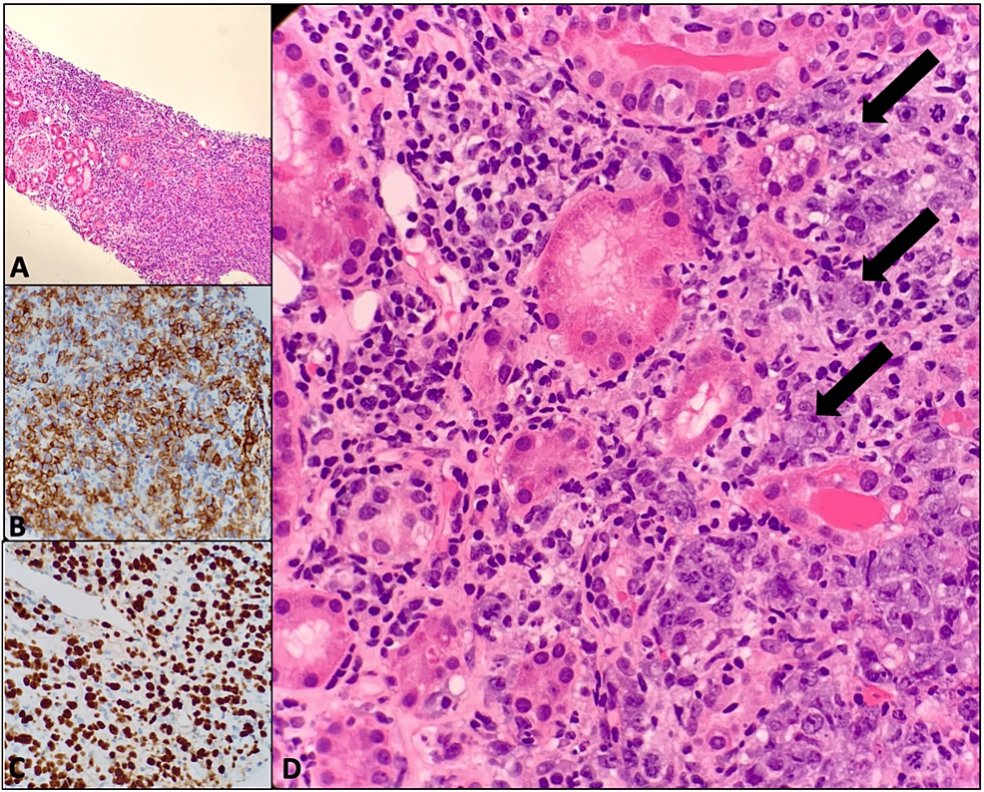
Retroperitoneal mass biopsy showing large B-cell lymphoma most consistent with diffuse large B-cell lymphoma involving the kidney Figure 2A shows large B-cell lymphoma involving renal parenchyma at 20x magnification. Figure 2B shows CD20 staining positive for large B-cell lymphoma cells. Figure 2C shows the Ki67 stain demonstrating a high proliferation index of large B-cell lymphoma cells consistent with the aggressive nature of the disease. Figure 2D shows 40x magnification of the involved renal tissue (arrows show lymphoma cells)

A 73-year-old man with no significant past medical history initially presented to the emergency room with left flank pain. A CT scan of the abdomen and pelvis at that time was significant for a 10.8 x 8.9 x 8.2 cm irregular heterogeneous mass arising out of the superior pole of the left kidney with extension to the undersurface of the spleen with celiac axis, retroperitoneal, and splenic hilum adenopathy (Figure 1). Biopsy of this mass revealed a non-germinal center subtype, double hit (BCL6 and MYC) HGBL, an aggressive subtype of diffuse large B-cell lymphoma (DLBCL, Figure 2). The patient was unable to complete a PET scan for staging. The initiation of the R-EPOCH chemotherapy regimen (rituximab, etoposide, prednisone, vincristine, cyclophosphamide, and doxorubicin) was planned for approximately six weeks after his initial presentation to the emergency room.

On arrival at the chemotherapy infusion center, the patient reported two weeks of increasing fatigue and shortness of breath as well as four days of melena, non-radiating epigastric pain that was worse after eating, and mild non-bloody nausea and vomiting. He was hemodynamically stable but found to be acutely anemic with a hemoglobin of 6.4 g/dL [mean corpuscular volume (MCV): 85 fL] from a baseline of 15 g/dL. He endorsed the use of non-steroidal anti-inflammatory drugs (NSAIDs) once a week for low back pain and denied smoking or alcohol use. He reported no personal or family history of GI malignancy. Recent testing had shown no evidence of cirrhosis or coagulopathy. He was administered two units of packed red blood cells with incrementation to a hemoglobin level of 8.3 g/dL and underwent urgent esophagogastroduodenoscopy (EGD).

**Figure 3:**
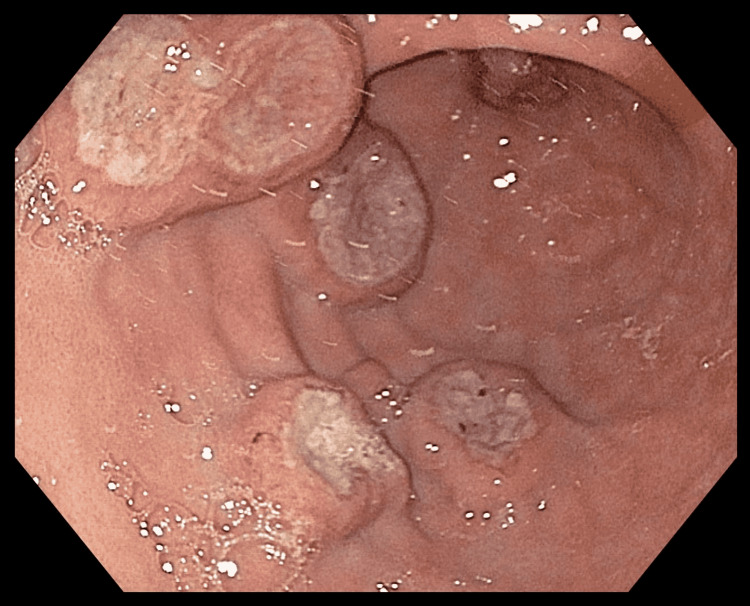
Endoscopic image of the gastric body showing large, discrete, raised, ulcerated, non-circumferential, and friable masses with heaped-up margins

**Figure 4:**
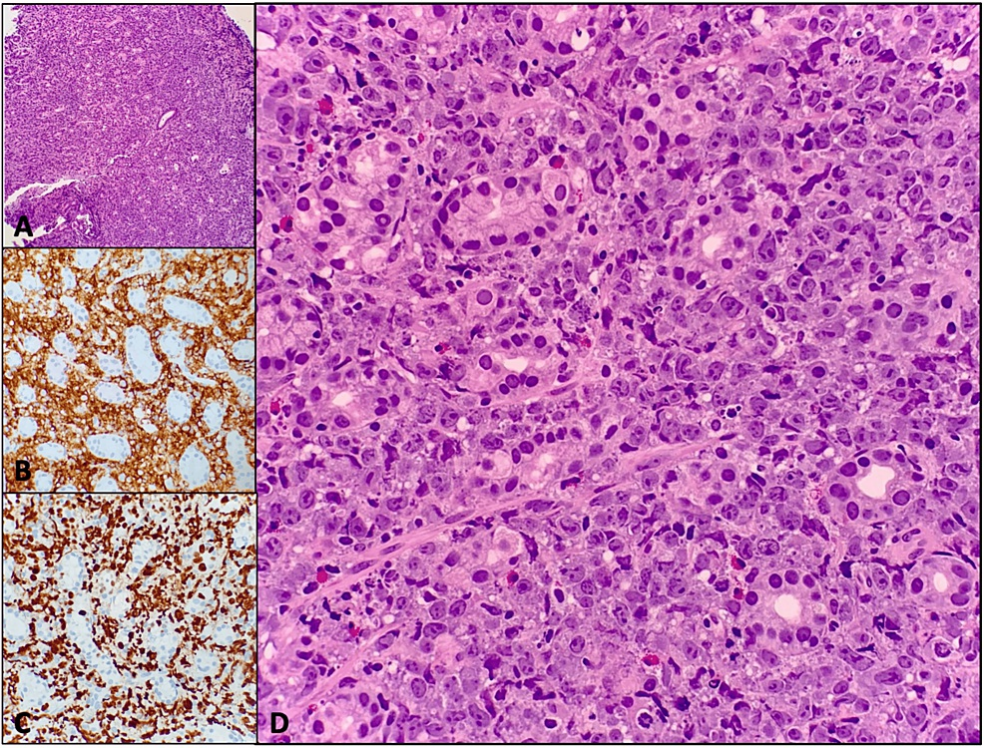
Gastric mass biopsy The images show submucosa infiltrated by intermediate to large lymphocytic cells with moderate cytoplasm, occasionally irregular nuclear contours, vesicular chromatin, and one or more small nucleoli, consistent with the patient's known high-grade B-cell lymphoma. Figure 4A shows large B-cell lymphoma involving gastric tissue at 20x magnification. Figure 4B shows CD20 staining positive for large b-cell lymphoma cells. Figure 4C shows the Ki67 stain demonstrating a high proliferation index of large B-cell lymphoma cells consistent with the aggressive nature of the disease. Figure 4D shows 40x magnification of the involved gastric tissue

**Figure 5:**
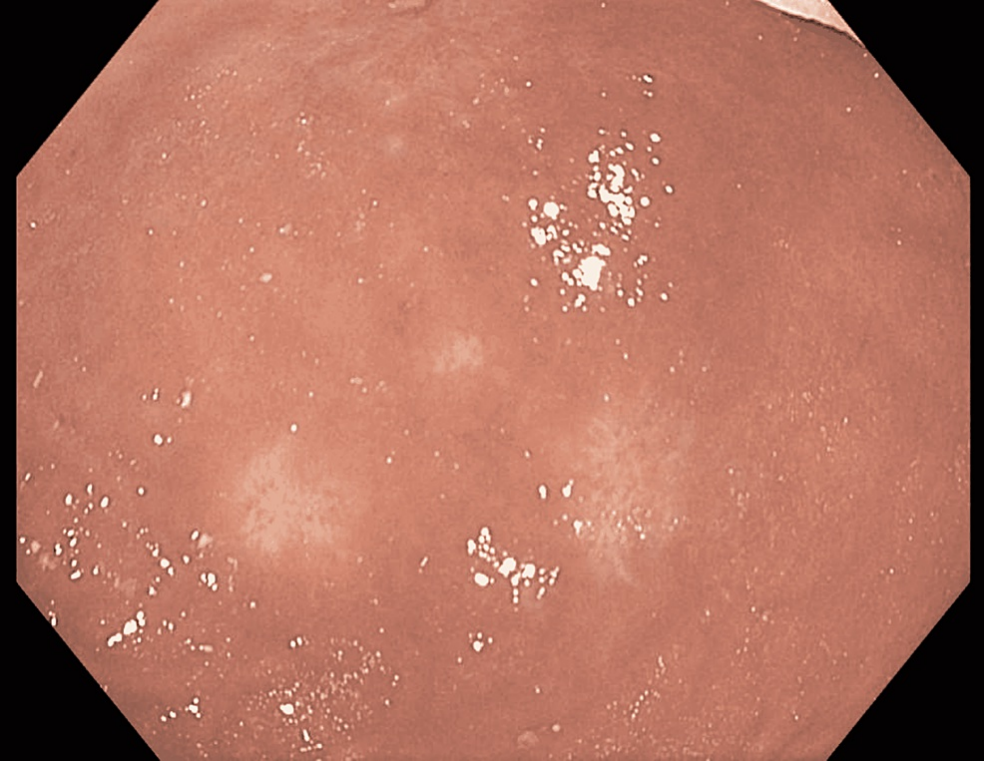
Endoscopy image of the gastric body following six cycles of R-EPOCH therapy A small, healed ulcer was found in the gastric body. The scar tissue was healthy in appearance. Histology was negative for malignancy R-EPOCH: rituximab, etoposide, prednisone, vincristine, cyclophosphamide, and doxorubicin

EGD revealed multiple (at least eight) large, discrete, raised, ulcerated, non-circumferential, and friable masses with heaped-up margins with bleeding on contact in the body and fundus of the stomach (Figure 3). Biopsies showed that the submucosa was infiltrated by intermediate to large lymphocytic cells with moderate cytoplasm, consistent with the patient's known HGBL (Figure 4). Staining for H. pylori was negative. The patient was continued on pantoprazole twice daily and initiated on R-EPOCH chemotherapy. Following the first cycle of chemotherapy, the patient had clinical resolution of UGIB as evidenced by no further melena and stable hemoglobin levels. A surveillance EGD was performed after six cycles of chemotherapy (approximately four months later), which showed normal gastric mucosa with mild scarring at the sites of prior lesions (Figure 5).

## Discussion

UGIB is a common and potentially life-threatening condition [1]. PUD is the most common cause of UGIB requiring hospitalization, accounting for approximately half of all cases [5,6]. However, rates of hospitalization due to PUD have declined over the last several decades whereas rarer causes, including esophagitis, Dieulafoy lesions, angiodysplasia, and malignancy, have been on the rise [7,8]. Malignancy is a rare cause of acute upper GI bleed, accounting for only 1-5% of cases, and is most likely due to primary gastric neoplasms [2-4]. Metastatic disease leading to UGIB is even rarer.

Our patient had been recently diagnosed with HGBL, a subtype of DLBCL with MYC and BCL2 and/or BCL6 rearrangements (he had MYC and BCL6 rearrangements) [9]. Mutations in these oncogenes are associated with aggressive disease as compared to other forms of DLBCL and are typically treated with more intensive chemotherapy regimens [10]. DLBCL encompasses a diverse class of diseases with various presentations, but it classically presents as a symptomatic mass in the abdomen, as seen in our patient. The prevalence rate of secondary GI involvement (e.g., metastatic disease) at the time of diagnosis is not known, but a previous study found that up to 60% of patients with advanced DLBCL have GI involvement [11]. Given the aggressive nature of our patient’s subtype of DLBCL (HGBL) and the adenopathy of the gastric axis lymphatic system, it is perhaps unsurprising that the stomach had metastatic disease. Additionally, given that the patient was unable to undergo a PET-CT, it is unclear whether the patient had asymptomatic gastric lesions at the time of initial evaluation that then rapidly progressed to symptomatic (e.g., bleeding) lesions when he presented for chemotherapy.

Endoscopic therapy is generally pursued in UGIB due to malignancy as a temporizing measure until definitive oncologic therapy is initiated [12,13]. However, endoscopic therapy in these patients has yielded poor outcomes, and GI malignancies causing severe UGIB are associated with a poor prognosis [3]. Initial hemostasis is achieved in 31-40% of cases, although some smaller studies have reported higher success rates [2,13]. Rebleeding is common with overall estimates of 41-80%, as compared to 8-24% in benign causes of UGIB [2,14,15]. Endoscopic therapy is technically challenging in these cases due to a variety of reasons including local vessel invasion, neovascularization of the tumor, and underlying coagulopathy [13]. There is no consensus on the modality of endoscopic therapy in malignancy-associated bleeding, largely due to the lack of large clinical trials and the heterogeneity of cases in the literature. Additionally, lymphomas of the GI tract are rarely associated with bleeding, and hence literature on malignancy-related GI bleeding is focused on primary GI malignancies such as gastric adenocarcinoma.

In our case, endoscopic therapy was not pursued as the patient was hemodynamically stable with appropriate incrementation in hemoglobin levels following blood transfusion. If the patient had decompensated, we would have considered hemostatic powder application due to its high success rate in achieving initial hemostasis, although 30-day rebleeding rates remain as high as 15% [16-19]. There is also a risk of hemostatic powder spray obstructing the target lesion if rebleeding occurs, thereby making other endoscopic therapies difficult [13,20]. In our case, this was viewed as an acceptable risk as the patient was planned for urgent chemotherapy as definitive treatment. Fortunately, our patient attained complete resolution of symptoms after a single round of chemotherapy and his metastatic lesions had completely regressed on subsequent EGD.

We recommend that metastatic disease be considered in patients with active malignancy who present with melena, especially if the primary malignancy has high metastatic potential such as melanomas or lymphomas. There are limited therapeutic options via endoscopy, and hence treatment should target the primary malignancy, typically via systemic chemotherapy, as was done in our patient.

## Conclusions

Metastatic disease should be considered in patients with active malignancy who present with melena, especially if the primary malignancy has high metastatic potential such as melanomas or lymphomas. Endoscopy should be performed for diagnosis. However, endoscopic therapeutic options remain limited. The treatment should target the primary malignancy, typically via systemic chemotherapy, as in the case of our patient.
